# The Influence of Physiological and Psychological Learning Mechanisms in Neurofeedback vs. Mental Imagery Against Binge Eating

**DOI:** 10.1007/s10484-020-09486-9

**Published:** 2020-09-29

**Authors:** Jennifer Schmidt, Alexandra Martin

**Affiliations:** 1 HSD Hochschule Döpfer University of Applied Sciences, Waidmarkt 3 & 9, 50676 Cologne, Germany; 2 Clinical Psychology and Psychotherapy, University of Wuppertal, Wuppertal, Germany

**Keywords:** Neurofeedback, Binge eating, Overeating, Treatment mechanisms, Electroencephalography

## Abstract

In biofeedback research, the debate on physiological versus psychological learning has a long tradition and is still relevant today, regarding new developments of biofeedback for behavior modification. Analyzing the role of these learning mechanisms may help improving the protocols and answer the question, whether feedback of physiological functions is necessary to modify a target behavior. We explored the presence and impact of physiological (EEG changes) versus psychological learning (changes in somatic self-efficacy) in a recently developed EEG neurofeedback protocol for binge eating. The protocol targets a reduction of food-cue induced cortical arousal through regulation of EEG high beta activity. In an experimental study accompanying a randomized controlled trial, pre and post treatment EEG measurements were analyzed in a neurofeedback group (*n* = 18) and an active mental imagery control group without physiological feedback (*n* = 18). Physiological learning in terms of EEG high beta reduction only occurred in the neurofeedback group. Post treatment, participants with successfully reduced binge eating episodes (≥ 50% reduction) showed lower EEG high beta activity than unsuccessful participants (*p* = .02) after neurofeedback, but not after mental imagery. Further, lower EEG high beta activity at post-treatment predicted fewer binge eating episodes in neurofeedback only. In mental imagery, somatic self-efficacy predicted treatment success instead of EEG activity. Altogether, the results indicate that physiological changes serve as a specific treatment mechanism in neurofeedback against binge eating. Reducing cortical arousal may improve eating behaviors and corresponding neurofeedback techniques should therefore be considered in future treatments.

“The spirit is willing, but the flesh is weak.” This proverb is frequently quoted at dinner parties or cafeteria buffets—that is, in a context of unwanted food consumption. Although we often try to resist temptations of palatable food, various factors, like stress, emotions or repeated food exposure, regularly boycott these intentions (Adam and Epel 2007; Haedt-Matt and Keel 2011; Swinburn et al. 2011). Under these circumstances, many people report food craving, a loss of control over eating restrictions, and binge eating episodes as a consequence (Boswell and Kober 2016; Stroebe et al. 2008). Repeated occurrences of binge eating episodes can result in weight gain (Dulloo and Montani 2015; Ozier et al. 2008) and are associated with body dissatisfaction, weight-related shame and guilt, distress, or depressive symptoms (Craven and Fekete, 2019; Presnell et al. 2004; Skinner et al. 2012). Providing individuals with enhanced capabilities to control bodily urges that facilitate dysfunctional eating behaviors is therefore an important objective in treatments for obesity, eating disorders, but also for general health behavior change.

Biofeedback (BF) treatments are traditional and well-approved means with the goal to strengthen control over somatic activity (Epstein and Blanchard 1977). Here, psychophysiological recordings are used to provide patients with external feedback on patterns in their physiological activity (Bagdasaryan and Le Van Quyen, 2013; Schwartz, 1976). Through the implemented feedback, it is possible to enable a person to control bodily responses by reinforcing individual strategies that result in desired physiological changes (Shapiro et al. 1964; Siniatchkin et al. 2000).

Given the relevance of loss of control in dysfunctional eating and the focus on control-processes in BF, it is not surprising that BF applications have recently emerged as promising treatments for dysfunctional eating behaviors, especially those that target neural activity (neurofeedback: NF) (for reviews, see: Bartholdy et al., 2013; Dalton et al., 2017; Imperatori et al. 2018). In their review, Imperatori et al. (2018) provided an overview of 13 studies (5 BF, 8 NF) that targeted eating disorders and related symptoms with feedback-based treatments. The review showed promising effects of these treatments and that recent studies increasingly reported physiological learning (i.e., treatment-related changes in physiological activity), for example in electroencephalographic (EEG) activity.

Despite of these observations on promising changes in eating-related outcome measures as well as physiological activity, one prominent old question is yet not satisfactorily answered in these BF-applications: Which role do physiological changes (i.e., changes in EEG activity) play in BF compared to psychological changes (i.e., increased self-efficacy and subjective self-control)? Moreover, which one of these two postulated mechanisms contributes more to target outcomes, such as changes in symptoms or behaviors via BF?

While physiological learning constitutes the central assumption of treatment mechanisms in BF (e.g., Schwartz 1976; Shapiro et al. 1964), the aforementioned psychological changes have become strongly advocated treatment mechanisms in this approach (Holroyd et al. 1984; Wickramasekera, 1999). Today, the analysis and comparison of *physiological* versus *psychological* learning in BF is still crucial to identify key treatment mechanisms, especially in novel applications and protocols (Gruzelier 2014a; La Vaque et al. 2002; Schwartz and Andrasik 2003; Sitaram et al., 2017). This is especially valid regarding the upcoming application field of dysfunctional eating, where physiological mechanisms and the contribution of changes in neural underpinnings still have to be explored (Dalton et al., 2017).

A detailed look at eating-related BF studies shows, that evidence for relevant physiological changes is mixed and dependent on the physiological target parameter. For example, Meule and colleagues (2012) used heart rate variability (HRV) BF and did not observe significant changes in HRV, despite of beneficial treatment effects on subjective food craving. Teufel and colleagues (2013) found an increase in eating-related self-efficacy and a reduction of sympathetic activity using electrodermal BF combined with food cue exposure. In real time fMRI-NF, Frank et al. (2012) observed changes in obese participants’ physiological regulatory abilities, yet without beneficial effects on eating behavior. Ihssen et al. (2017) found reduced reward-related brain activation in a food cue-exposure real time fMRI-NF, but no effects on food craving. With regard to EEG-NF, Lackner et al. (2016) reported changes in resting-state EEG theta activity in a NF treatment targeting alpha activity in patients with anorexia nervosa. They also reported improvements in eating-related variables (e.g., dietary restrictions) but not in weight status or body image. Imperatori et al. (2017) observed increased EEG alpha activity after alpha/theta NF against food craving, as well as reductions in subjective food craving. Finally, Leong et al. (2018) reported reductions in food craving and physiological learning in an infraslow EEG-NF treatment.

In two randomized controlled trials (RCTs), we evaluated a ten-session NF treatment to reduce overeating and binge eating episodes (Schmidt and Martin, 2015; 2016). The rationale of these studies based on cue exposure with palatable food cues and subsequent down-regulation of dysfunctional EEG high beta activity (23–28 Hz) that is associated with tense arousal, craving, disinhibition, and dysfunctional eating behaviors. Changes in this brain activity range have been shown to be associated with exposure to appealing cues in eating behavior and addiction research (for reviews, see: Blume et al., 2019; Parvaz et al. 2011). While EEG high beta activity has been added as a supplementary spectral range to control hyperarousal (e.g., Egner and Gruzelier, 2001; Keith et al. 2015; Rostami et al. 2012), it has seldom been the main target of regulation in NF protocols. The few studies using EEG high beta as a target range found positive results regarding the presence and influence of physiological learning in this spectral range (Paquette et al. 2009; Zotev et al. 2014). Still, none of these studies so far had targeted eating behavior.

While both of our studies on clinical outcomes (Schmidt and Martin, 2015; 2016) showed efficacy of NF in reducing overeating and binge eating, the NF-training system used in the two studies did not allow for a post-hoc analysis of changes in EEG activity associated with the treatment. Therefore, the second RCT (Schmidt & Martin, 2016) was accompanied by an experimental psychophysiological laboratory study. The experiment served to assess NF-associated EEG changes from pre to post treatment in a high beta protocol and their associations with self-reported treatment-outcomes. In the present study, we assessed EEG activity prior to the first treatment and the last treatment session in a separate assessment, using experimental food cue exposure followed by self-regulation without feedback to shed light on potential mechanisms involved in treatment outcomes. The following research questions are addressed:

Does the NF treatment result in physiological learning?

## H1:

Does physiological learning differ between successful (≥ 50% binge eating reduction) and unsuccessful (< 50% binge eating reduction) participants in NF at post-treatment?

## H2:

At post-treatment, successful participants in NF show lower levels of EEG high beta activity during self-regulation phases after cue exposure than unsuccessful participants.

Does physiological learning show stronger relations to post-treatment outcomes than psychological learning in NF?

## H3:

Post-treatment EEG high beta activity predicts binge eating episodes more strongly than subjective somatic self-efficacy in NF.

For a comparison of possible effects that might have been caused by unspecific treatment effects (e.g., relaxation, therapeutic relationship, and general repeated participation in a treatment) we additionally investigated changes of the physiological and psychological variables in an active control group, training with a mental imagery (MI) protocol, without any feedback on their physiological activity. This analysis bases on a merged-groups-sample of the RCT (Schmidt and Martin 2016).

## Method

### Study Design

The study is based on a pre-post design in two active intervention groups. Data were obtained in an additional experimental study during an RCT that examined the specific efficacy of a NF training to reduce binge eating episodes in female restrained eaters compared to two control groups (Schmidt and Martin 2016). In contrast to the published study, the present study based on the additional experimental study in the merged-groups-sample of the RCT. This sample includes participants from the original NF and MI groups as well as former waitlist-participants, who participated in NF or MI treatments after the waiting period. All of them participated in sessions of this experimental study prior to the first and last session of their active treatment phases.

We assessed EEG activity and self-reports on binge eating episodes, as well as somatic self-efficacy prior to the first (T0) and final (T1) treatment sessions. Before participation, all participants were informed on the experimental procedures, including randomization, physiological measurement, data handling, and treatment protocols. All participants included in the study provided written informed consent. The ethics committee at the University of Wuppertal approved the research protocol.

### Sample

The sample consisted of adult female participants, screened as restrained eaters (values ≥ 12; German version of the *Restraint Scale*, Dinkel et al. 2005) who reported regular occurrences of binge eating episodes. Women priorly diagnosed with (or positively screened for) clinical eating disorders, insulin-dependent diabetes mellitus, any neurological or severe mental disorders were excluded from the study. Further exclusion criteria encompassed regular use of medication associated with weight fluctuations, alcohol dependency, pregnancy, and adherence to a time limited weight-loss diet (e.g., formula diets). We recruited participants with media reports and flyers in medical practices. Eligibility for participation was assessed via online questionnaire during recruitment.

A total of 123 persons were screened for eligibility, whereof 48 were either not eligible to participate (*n* = 17) or did not respond our invitation to an information session on the study (*n* = 31). A blinded and uninvolved person then randomly assigned the remaining 75 subjects to either NF treatment, MI treatment, or a waitlist group (*n* = 25 each). Throughout the first study phase, 16 women discontinued the study (see also Schmidt and Martin 2016).

**Fig. 1:**
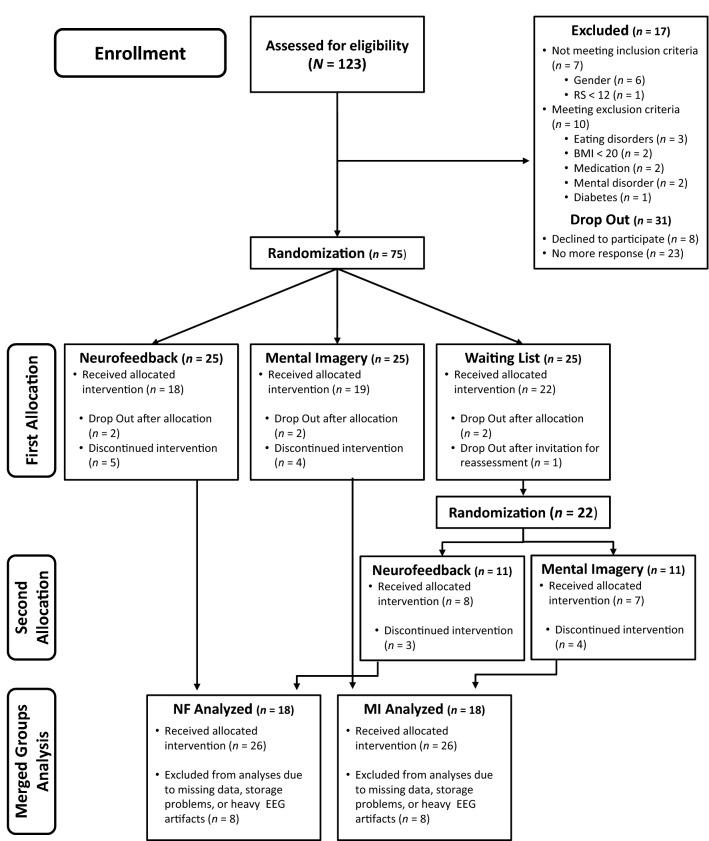
Participant flow according to CONSORT guidelines

Waitlist participants were randomly assigned to one of the two active treatments after an 8 week waiting period (*n* = 11 each). In the second study phase, *n* = 7 women dropped out. The resulting merged groups sample (*n* = 26 in NF and MI resp.) served as the target sample for the present EEG study. Here, some participants (*n* = 8 in NF and MI respectively) had to be excluded from statistical analyses due to storage problems, bad signal quality, or heavy artifacts in EEG recording, resulting in a final sample of *n* = 18 for each group (for participant flow, see Fig. 1).

### Procedure

We conducted experimental and treatment sessions between April and October 2014. Two calm and highly comparable training rooms, as well as the NF equipment and psychophysiological recordings devices were provided by *psyrecon GmbH* (Wuppertal, Germany). According to the scope of this study, descriptions will focus on the experimental study and only give a brief overview on the treatments from T0 to T1. For a more detailed description of the treatment procedures, see Schmidt and Martin (2016).

#### Experimental Sessions

At T0 and T1, all women attended the individual experimental sessions, including psychophysiological recording during food cue exposure and in subsequent self-regulation. They were asked not to eat for three hours prior to the sessions, to ensure appeal of the selected food cues. During the sessions, participants sat in a comfortable armchair in 1 m distance of a 22″ flat screen. The screen displayed the experimental presentations by using standardized presentations, yet with personalized food cues in *MS PowerPoint*.

All women first filled in a questionnaire booklet containing the target instruments (see “Assessment instruments” section). Experimenters then attached EEG electrodes after corresponding preparation (see “Physiological recording and analysis” section). When signal quality was satisfactory, the presentation was started, displaying a standard instruction in black letters on a white background. Participants were informed about the procedure and duration of alternating cue exposure and self-regulation phases. They were instructed to avoid movements or speaking, and to keep their eyes open during the presentation.

For cue exposure phases (T0 & T1), participants had to imagine the displayed foods as vividly as possible, including smell, taste, and consistency, which has previously been evaluated as a successful strategy to induce craving (Sobik et al. 2005). During self-regulation phases at T0, participants should relax the way they would usually relax with open eyes; for self-regulation phases at T1, participants used the strategies learned in the treatments (NF or MI).

After the instruction, physiological recording started and was synchronized with the presentation. A baseline recording was performed for 120 s. Then, three alternating phases of cue exposure (30 s each) and self-regulation (120 s each) succeeded. In cue exposure phases, participants were confronted with three individually selected, appealing digital pictures of foods they regularly crave and binge on. These pictures were also used during the corresponding treatments. In self-regulation phases, an animated landscape of a beach at sunset was displayed, which was also used in the treatment sessions. However, the EEG system did not provide any feedback on brain activity during the experimental sessions. Altogether, the experimental sessions lasted 9.5 min and contained the same stimuli (pictures, animation) at T0 and T1.

#### Physiological Recording and Analysis

We obtained physiological data using the *Varioport Biosignal Recorder* (*Becker MediTec*) and the *Variograf* software. Besides EEG recording, galvanic skin response and heart rate were assessed for another research project. Results will be presented elsewhere. EEG was derived with an active and pre-amplified, unipolar 5-channel EEG device (Ag/AgCl electrodes) with reference and ground electrodes on the right and left mastoid. Recording sites were Cz, Fz, F3, F4, and Pz, according to the international 10–20 system (Jasper 1958). We used flexible EEG caps (*EasyCap*) to attach electrodes. Skin preparation was conducted with abrasive *One-Step EEG* peeling paste and 65% isopropyl alcohol to ensure satisfactory impedance levels. To retain skin contact and sufficient conductivity, we used *SuperVisc* (*EasyCap*) electrode paste for active EEG recordings.

After electrode attachment, the experimenter checked signal quality and adjusted electrodes whenever signals were not satisfying or impedance levels were too high. Additionally, for correction of ocular artifacts, we acquired a vertical electrooculogram (EOG) via a 2 mm Ag/AgCl electrode and conductivity enhancing electrode paste (*Electrode Cream, GE Medical Systems*) below the left eye. Analogue sampling rate was 1024 Hz. A 50 Hz notch-filter was included in the recording device. During experimental recordings, the experimenters monitored the signals and protocolled visible muscular artifacts or decreasing signal quality.

Analysis of EEG data was performed offline, using a *MatLab* based tool (programmed by Prof. Dr. Bertrand Massot, *INSA Lyon*), to perform Fast Fourier Transformation and obtain spectral power of the relevant EEG frequency ranges. The method implied bases on a shifting window over 10 s-segments without overlap throughout the course of the experimental session. It uses the Welch periodogram (Welch 1967) due to the advantage of being independent of predetermined window size. A rectangular window was applied to analyze spectral power in the whole range of EEG frequencies from 1 to 30 Hz. Correction of ocular artifacts was performed based on the EOG recordings, using principal component analysis (PCA) as the superior method for automatic corrections, avoiding spectral distortions (Wallstrom et al. 2004).

We exported calculated values (absolute Power, µV^2^) as data sheets and screened them for artifacts protocolled during the experimental sessions. This is especially important because the frequency range of interest, EEG high beta activity (23–28 Hz) may be influenced by muscular activity due to overlapping frequency ranges (Muthukumaraswamy 2013). Values were then averaged for each electrode position and every separate 10 s-interval over the spectral ranges of interest (delta: 1–3 Hz; theta: 4–7 Hz; alpha: 8–12 Hz; sensorimotor rhythm [SMR]: 13–15 Hz; low beta: 16–22 Hz; high beta 23–28 Hz). To calculate an EEG power indicator for statistical analyses of the current research questions, we determined mean values over all three 120 s self-regulation phases after cue exposure at T0 and T1. Whenever artifacts only affected single 10 s-intervals, values were replaced by mean values in the respective phase. Participants with more than five 10 s-intervals affected by artifacts (i.e., more than 15% of the recording) or those who showed decreasing signal quality during the sessions were excluded from analyses (*n* = 16).

Since absolute spectral power values in EEG recording can vary heavily between participants and between repeated measurements, relative spectral power was calculated dividing absolute power for each target frequency (e.g., high beta in µV^2^) by the overall sum of absolute power of the spectral ranges (delta to high beta in µV^2^). Decimal values (ranging from 0 to 1) were then transformed to percentages. Due to the potential effects of the NF training on baseline EEG activity from T0 to T1 (Gruzelier 2014a), we did not perform any baseline corrections to avoid neglecting those possible outcomes. Only values of the electrode site which was used as a training position in NF (Cz) will be reported in the present paper.

#### Treatments

Both treatments—NF and MI—consisted of ten sessions based on standardized treatment manuals (see Schmidt and Martin 2016 for further details). Each session began with a 180 s adaptation phase. Then participants in both groups were repeatedly exposed with individual pictures of foods, which regularly induce craving and binge eating (ten exposures, 30 s each). Participants should imagine the foods as vividly as possible. Each exposure phase was followed by 120 s of the self-regulation task.

For the NF group, the self-regulation task was the down-regulation of EEG high beta activity (23–28 Hz) as a means to reduce cortical arousal caused by cue exposure to appealing food cues (Blume et al. 2019; Parvaz et al. 2011). The signal was derived from a unipolar online EEG assessment (*Mindfield Mindmaster EEG*) at the vertex position (Cz), with reference and ground electrodes on the earlobes. Feedback on EEG high beta activity was displayed as bar diagrams to be kept below a threshold as well as through a beach landscape animation. Activity below thresholds was rewarded (green bar, fluent animation); Activity surpassing thresholds was inhibited (red bar, stopping animation). An additional bar diagram indicated artifacts caused by eye-movements or muscular activity (e.g., swallowing or body movements) during the sessions. In addition, the NF-trainers monitored the signal quality over the course of the sessions.

Participants were encouraged to try different strategies for self-regulation (e.g., thinking of nothing, thinking of colors, using mantras or imagery) throughout the first treatment sessions. They were instructed to pursue the ones that were rewarded by the NF system. At the beginning of the sessions, thresholds were set to 4 μV for EEG high beta and 1–1.5 μV for artifacts. During the adaptation phase of each session, the trainers adjusted the thresholds to the individuals’ baselines to match predefined success rates. These success rates were reduced over the training course (stepwise: 85% to 70%). In cases of strong deviations from the thresholds over the course of one training session for more than 5 min, the trainers slightly adjusted the thresholds to avoid discouragement of the participants. After ten exposure phases, we additionally instructed participants to upregulate alpha activity (8–12 Hz) for the concluding 180 s. We selected this procedure to assure that participants conclude the sessions in a relaxed state of mind (Gruzelier 2014b). Each session lasted approximately 45 min, including preparation.

For the MI group, participants were made familiar with the mental imagery approach (Kemps and Tiggemann 2007; Knäuper et al. 2011), which incorporates vivid imagination of pleasant, relaxing, and food-unrelated mental images (e.g., sitting by the sea or walking through beautiful landscapes). Through this procedure, a state of relaxation should be induced. Alternative imagery should replace craving related food imagery by claiming visuospatial working memory capacities. To find the most suitable mental image, all women should try different image contents and observe which image would fulfill the prerequisite of being easy to retrieve, relaxing, and vivid. Participants then visualized this image in every self-regulation phase. In all sessions, a visual beach animation was fluently presented to assist relaxation. After ten exposure phases, we additionally instructed participants to relax with their eyes closed for 180 s to end the sessions in a relaxed state of mind. Each session lasted approximately 35 min.

### Assessment Instruments

#### Screening Instruments

For screening purposes regarding inclusion and exclusion criteria, we assessed age, gender, Body Mass Index, current dieting status, medication, histories of eating disorders, alcohol abuse, neurological and mental disorders, and diabetes online. Further, we used the *Restraint Scale* (RS; Dinkel et al. 2005) with a ten item cut-off sum score ≥ 12 to determine restrained eating, and the German *Eating Disorder Examination Questionnaire* (EDE-Q; Hilbert and Tuschen-Caffier 2006) with a 22 item cut-off mean score < 4 (Mond et al. 2006) to determine disordered eating. For both measures, good psychometric properties have been reported (Dinkel et al. 2005; Hilbert and Tuschen-Caffier 2006).

#### Binge Eating Episodes

We assessed the frequency of binge eating episodes with a questionnaire (Schmidt and Martin 2015), asking participants to retrospectively rate the number of binge eating episodes within the last seven days. The rating scale was preceded by a definition of binge eating episodes in the subclinical context of this study, defining them as being induced by food craving urges and resulting in undesired consumption of high calorie food without physiological hunger. The reported number of binge eating episodes was used as an indicator of binge eating frequency at T0 and T1. To separate successful and unsuccessful participants for subgroup analyses, a criterion of at least 50% symptom reduction in binge eating from T0 to T1 was regarded as clinically relevant success, in line with previous suggestions (e.g., Blanchard and Schwarz 1988).

#### Somatic Self-efficacy

We assessed somatic self-efficacy with a five item questionnaire on the perceived ability to control bodily responses and to relax (e.g., “I am able to control my bodily reactions”; “For me, it is easy to calm down when I am upset”) with 7-point answer scales (0 = *do not agree at all*; 6 = *fully agree*). The mean score served as an indicator of somatic self-efficacy. Internal consistency of the questionnaire was acceptable, α = 0.70.

### Statistical Analyses

Some data distributions violated normality assumptions. We therefore used non-parametric methods for group comparisons and backed up regression analyses with bootstrapping techniques.

To analyze possible reductions in relative spectral EEG high beta activity throughout self-regulation phases at T0 and T1, we performed separate within-groups Wilcoxon-tests for the NF and MI group. To provide an overall picture of EEG activity during self-regulation at T0 and T1, we conducted additional exploratory pre-post comparisons for the other analyzed EEG spectral ranges (delta, theta, alpha, SMR, and low beta) apart from hypothesis testing. We used the same statistical procedure for somatic self-efficacy to account for comparable effects. To address the question whether successful and unsuccessful participants differ in their amount of relative spectral EEG high beta post-treatment, we used Mann–Whitney *U*-tests for either group.

We conducted hierarchic regression analyses to determine, whether EEG high beta activity or somatic self-efficacy would predict binge eating episodes at post-treatment. EEG high beta activity was first inserted as predictor for NF (model 1), followed by the addition of somatic self-efficacy (model 2). We then tested the same model in the MI group. To back up regressions, we used a bootstrapping procedure (*n* = 1000). Significance levels were determined at *p* < 0.05, one-sided for H1 and H2, and two sided for H3.

In line with recommendations (Fritz et al. 2012), effect sizes for H1 and H2 were calculated as *r* based on *Z*-values due to partly skewed data. Effect sizes for H1 were calculated as $r=\left|\frac{z}{\sqrt{2n}}\right|$. Effect sizes for H2 were calculated as $r=\left|\frac{z}{\sqrt{N}}\right|$. For *r*, values ≥ 0.50 indicate large effects, values ≥ 0.30 indicate medium effects, and values ≥ 0.10 indicate small effects (Fritz et al. 2012).

## Results

We did neither observe any significant differences between groups in demographic or screening variables (see Table 1), nor in any outcome variable at pre-treatment (all *p*s > 0.121), indicating comparable groups.

**Table 1:** Demographic and screening data of the analyzed sample

Variable	Neurofeedback (*n* = 18)	Mental imagery (*n* = 18)	Total (*n* = 36)	Test statistics
	*M* (*SD*)	*M* (*SD*)	*M* (*SD*)	
Age	47.94 (14.24)	39.22 (14.75)	43.58 (14.96)	*U* = 211.5, *p* = .121
Body Mass Index	27.89 (4.93)	27.26 (4.86)	27.58 (4.84)	*U* = 170.0, *p* = .812
Restraint score	19.39 (4.39)	19.28 (3.97)	19.33 (4.13)	*U* = 158.0, *p* = .911
Eating pathology (EDE-Q total)	2.19 (0.91)	2.39 (1.09)	2.29 (0.99)	*U* = 133.5, *p* = .376

Addressing the first research question, we found that NF participants showed significantly reduced EEG high beta activity during self-regulation phases after cue exposure at T1 compared to T0 (*p* = 0.027, medium effect). This effect was not observed for MI participants (*p* = 0.142, small effect). There were no significant differences in any other exploratory T0-T1 comparisons of EEG activity. Descriptive data and test statistics are displayed in Table 2.

**Table 2:** Group EEG and self-report data pre and post treatment

	Pre-treatment		Post-treatment		Test statistics		
	*M*	(*SD*)	*M*	(*SD*)	Wilcoxon	*p*	*r*
*Neurofeedback* (*n* = 18)							
Somatic self-efficacy	2.93	(0.91)	3.79	(0.81)	6.5	< .001**	.58
EEG high beta %	4.68	(2.97)	3.19	(1.32)	130.0	.027*	.32
EEG low beta %	7.34	(4.21)	6.27	(2.93)	114.0	.115	.21
EEG SMR %	7.97	(3.86)	8.12	(3.63)	83.0	.466	.02
EEG alpha %	13.80	(6.96)	15.85	(6.96)	53.0	.084	.24
EEG theta %	16.89	(5.00)	17.79	(4.67)	57.0	.115	.21
EEG delta %	49.32	(16.43)	48.80	(11.87)	100.0	.276	.11
Binge eating episodes	4.38	(2.77)	3.27	(3.34)	105.5	.027*	.33
*Mental imagery* (*n* = 18)							
Somatic self-efficacy	2.71	(0.89)	3.41	(1.06)	16.5	.006**	.47
EEG high beta %	4.04	(2.57)	3.39	(2.06)	111.0	**.142**	.19
EEG low beta %	7.01	(2.80)	6.18	(3.11)	119.0	.077	.24
EEG SMR %	8.85	(3.70)	8.44	(4.42)	97.0	.320	.08
EEG alpha %	16.18	(6.61)	18.12	(8.32)	73.0	.305	.09
EEG theta %	18.39	(3.64)	17.62	(3.19)	113.0	.123	.20
EEG delta %	45.53	(10.50)	46.24	(13.28)	87.0	.483	.01
Binge eating episodes	4.50	(3.84)	2.83	(3.49)	120.0	.020*	.35

Test statistics: within-groups *t*-tests; *p*-values: one-sided, * *p* < .05; ** *p* < .01. Conventions for effect size *r*: *r* ≥ .10 small effect; *r* ≥ .30 medium effect; *r* ≥ .50 large effect. For *p*-values of group-comparisons used for hypothesis testing (H1, bold print) are corrected for multiple comparisons (Holm); *p*-values of all other exploratory comparisons are reported without corrections for multiple testing

**Fig. 2:**
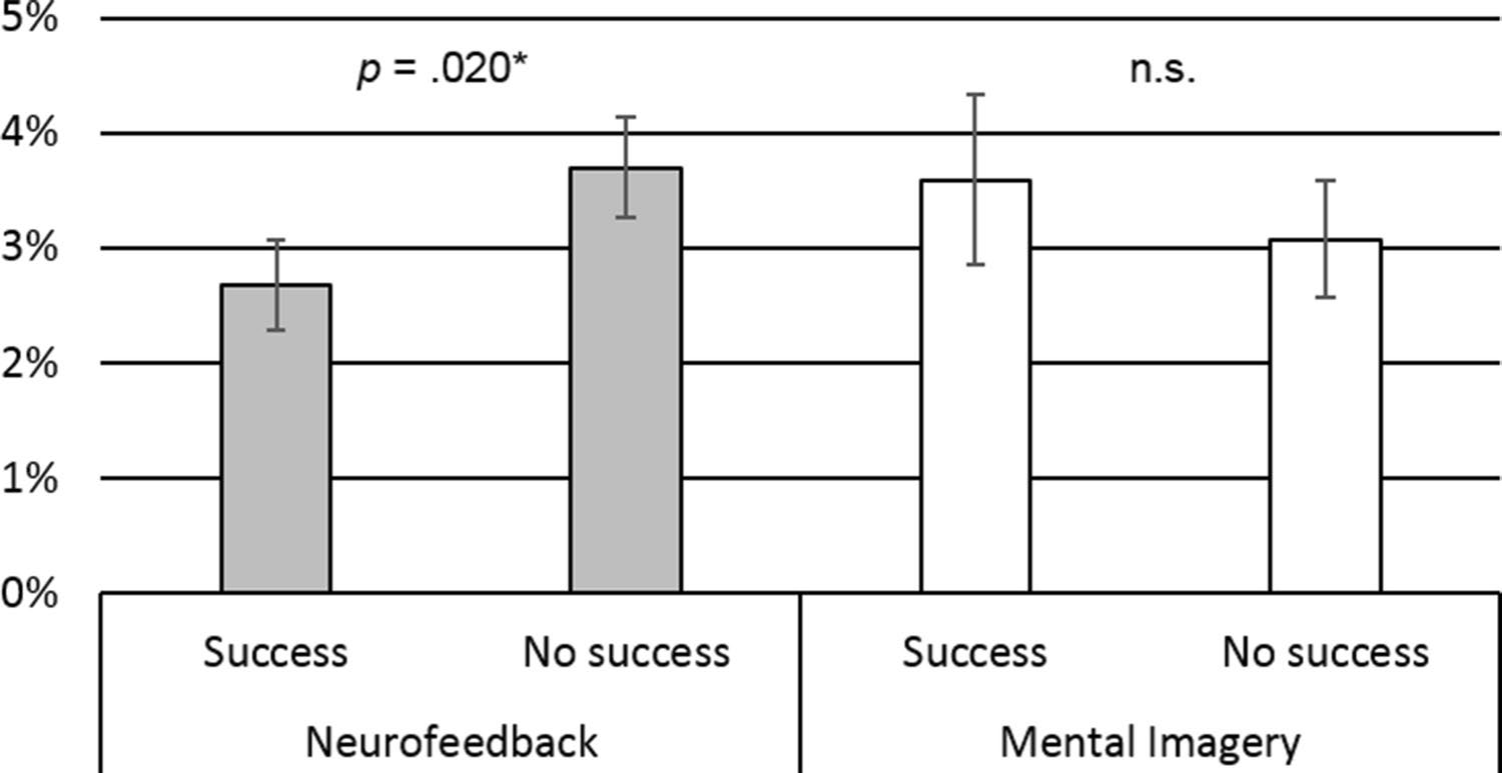
Comparison of post-treatment EEG high beta activity (relative) in participants with or without clinically relevant success (≥ 50% vs. < 50% symptom reduction); Test statistics: Mann–Whitney U-Test, error bars indicate standard errors, *p < .05

For the second research question, groups were divided into subgroups of participants with successful (NF: *n* = 9, MI: *n* = 11) and non-successful (NF: *n* = 9, MI: *n* = 7) treatment outcomes based on at least 50% reductions in weekly binge eating. At T1, successful NF participants had significantly lower EEG high beta activity (*M* = 2.67%, *SD* = 1.18%) compared to unsuccessful NF participants (*M* = 3.70%, *SD* = 1.31%), *Z* = − 2.08, *p* = 0.020, *r* = 0.50 (large effect). This difference cannot be attributed to initial EEG high beta activity, as at T0, no difference was observed, *Z* = − 0.84, *p* = 0.218, *r* = 0.20. Further, no effect was found comparing successful (*M* = 3.59%, *SD* = 2.44%) and unsuccessful (*M* = 3.07%*, SD* = 1.36%) participants in MI, T1: *Z* = 0.23, *p* = 0.430, *r* = 0.05; T0: *Z* = 0.50, *p* = 0.330, *r* = 0.12. Results are depicted in Fig. 2.

Hierarchic regression analyses for the third research question showed that in NF, model 1, with EEG high beta activity as a predictor, explained 21% of the variance in binge eating at post-treatment. When somatic self-efficacy was added as a predictor in model 2, the amount of variance explained increased to 34%. While EEG high beta activity remained significant as a predictor, somatic self-efficacy only showed a trend towards significance as a predictor, yet became significant in the bootstrapped model (*p* = 0.046). Statistical details for both models are shown in Table 3.

**Table 3:** Hierarchic regression for the prediction of binge eating episodes after Neurofeedback

Variable	*B*	β	*t*	*p*	*B*: CI 95%	Δ*R*^*2*^
*Model 1*						
Constant	− 0.82		− 0.44	.699	[− 4.79; 3.16]	
EEG high beta T1	1.29	.51	2.36	.032	[0.13; 2.44]	.26
*R*^2^_adj_ = .21; *F*(1, 16) = 5.55; *p* = .032						
*Model 2*						
Constant	5.10		1.52	.149	[− 2.05; 12.25]	
EEG high beta T1	1.41	.56	2.82	.013	[0.34; 2.48]	.26
Somatic self-efficacy T1	− 1.67	− .41	− 2.05	.058	[− 3.41; 0.07]	.16
*R*^2^_adj_ = .34; *F*(2, 15) = 5.43; *F* change = 4.21; *p* = .017						

Y = Binge eating episodes post treatment, *n* = 18, T1 = post-treatment

For MI, the regression model using EEG high beta activity as a single predictor (model 1) was not significant (*p* = 0.785) and did not explain any variance in post-treatment binge eating. However, when somatic self-efficacy was added as a predictor (model 2), the model explained 32% of the variance in post-treatment binge eating. The bootstrapped model confirmed the result pattern. Statistical details for both models are shown in Table 4.

**Table 4:** Hierarchic regression for the prediction of binge eating episodes after Mental Imagery

Variable	*B*	β	*t*	*p*	*B*: CI 95%	Δ*R*^*2*^
*Model 1*						
Constant	2.44		1.46	.163	[− 1.09; 5.96]	
EEG high beta T1	0.12	.07	0.28	.785	[− 0.78; 1.01]	.01
*R*^2^_adj_ = − .06; *F*(1, 16) = 0.08; *p* = .785						
*Model 2*						
Constant	9.77		3.61	.003	[3.99; 15.54]	
EEG high beta T1	0.04	.02	0.10	.920	[− 0.69; 0.76]	.01
Somatic self-efficacy T1	− 2.07	− .63	− 3.11	.007	[− 3.48; − 0.65]	.39
*R*^2^ _adj_ = .32; *F*(2, 15) = 4.91; *F* change = 9.69; *p* = .023						

Y = Binge eating episodes post treatment, *n* = 18, T1 = post-treatment

## Discussion

The present study aimed at investigating the presence and role of physiological learning mechanisms (i.e., reductions in EEG high beta as a marker of cortical arousal) versus psychological learning mechanisms (i.e., enhancement of somatic self-efficacy and abilities to relax) in a NF treatment against binge eating. We aimed at analyzing, how these learning processes relate to treatment success, and if physiological changes are specific for NF. Therefore, we conducted the same analyses in a group training with a MI treatment in a corresponding setup with imagery-related self-regulation after food cue exposure.

The results indicate the presence of physiological learning in NF: Participants in NF were able to reduce their cortical arousal—as measured by EEG high beta activity − from pre- to post-treatment, confirming H1. In comparison, we did not observe EEG high beta reductions in MI. Hence, these physiological changes seem to be specific for NF. Enhancements in somatic self-efficacy were observed in both treatments, indicating that the reduced EEG arousal in NF is not only caused by subjective relaxation (e.g., Kim et al. 2014).

We further obtained results that mark the relation between a reduction in cortical arousal and treatment success: Post-treatment EEG high beta activity differed among successful and unsuccessful participants, with lower EEG high beta activity in successful participants, confirming H2. This effect not observed for MI.

After the treatment, lower EEG high beta activity predicted the frequency of binge eating episodes. While post-treatment somatic self-efficacy did exert some influence in NF, the impact of EEG high beta activity was larger, confirming H3. In comparison, EEG high beta activity was not related to treatment outcomes in MI.

In present sample, the frequency of binge eating episodes decreased in both treatment groups. In the prior RCT sample (Schmidt & Martin, 2016), the NF group showed less frequent binge eating episodes post-treatments compared to a waitlist, while the MI group did not. Still, a within group comparison at follow-up showed significant reductions of binge eating in both active groups, which is confirmed in the present analysis. However, the present analysis shows that the two treatments seem to work on different pathways: Although perceived subjective self-regulatory abilities play a certain role in NF —which is in line with previous findings in BF research (Holroyd et al. 1984; Wickramasekera 1999) —physiological learning still showed a greater influence in this treatment type. In MI, only psychological learning, i.e. somatic self-efficacy accounted for improvements in binge eating.

One important prerequisite of the NF and BF approach is the view that physiological activity associated with dysfunctional states or behaviors is altered, to change the behavior or state itself consequently (Niv 2013; Schwartz 1976). Critiques have recently again challenged this view, pointing out that treatment effects in NF may be attributed to unspecific treatment factors (Thibault et al. 2016), placebo effects (Thibault et al. 2017) or ‘neuroenchantment’ (i.e., enhanced credibility of studies or treatments that use brain imaging; Ali et al. 2014). The emerging amount of studies that explicitly address and analyze physiological learning in BF-treatments (Imperatori et al. 2018) will help to specify the relevance of these placebo effects. While the present study tried to control for unspecific treatment effects by comparing the influence of learning processes in two active treatments, the effect of neuroenchantment caused by the technical equipment used in the NF-group needs further investigation. Future studies should therefore also include sham-feedback—preferably in double-blind designs (Ros et al. 2020)—including groups that train with the same technical setup to assess the specific contribution of neural regulation (Thibault et al. 2016). However, such studies should also assess possible nocebo effects that might be caused by false feedback (Colloca and Miller 2011). Besides these methodological suggestions, the scientific rigor of RCTs on NF treatments should further be improved by preregistration of NF studies to prevent false-positive results and under-reporting of negative outcomes in NF research (Ros et al. 2020; Thibault et al. 2018).

Still, according to the results of the present study, physiological changes seem to play a role in this NF protocol against binge eating, linking reduced cortical arousal to less craving and less frequent binge eating episodes (Blume et al. 2019; Parvaz et al. 2011). This finding is also important because EEG high beta activity has seldom been the main target frequency range in NF protocols (Paquette et al. 2009; Zotev et al. 2014), but has instead mostly been used as a supplementary control range (Egner and Gruzelier 2001; Keith et al. 2015). Our findings indicate that high beta is a trainable frequency range that can be targeted in NF when psychological correlates are indicative for this procedure. However, due to different classifications of ranges of the beta band in the EEG, we have to mention that the present study trained high beta in the range of 23–28 Hz only. Researchers should therefore be cautious in transferring the present findings on physiological learning to other protocols with different classifications of the beta range (e.g., general beta activity, 13–30 Hz).

Despite additionally training EEG alpha activity at the end of the NF sessions, pre-post changes in alpha activity were not significant. However, given the treatment setup and instructions, this result is not surprising: Participants only had to upregulate alpha activity in the last 180 s of each sessions and not as a self-regulation task after the food cue exposure. Thus, the specific alternations in high beta activity further point into the direction that pre-post changes could be attributable to the self-regulation-training effects in NF, rather than to general relaxation.

Still, a discussion of limitations is warranted. Statistical power is reduced due to missing EEG data that had to be excluded because of artifacts. Although we tried to limit constraints exerted by sample size with appropriate statistical analyses, the study should be replicated with larger samples.

The present study allowed assessing changes in EEG activity from the start to the end of the treatment. However, we were not able to determine the physiological learning curves for NF based on the course of EEG changes within every session and between sessions because of technical constraints of the training system. Especially within-session learning is a parameter, which is frequently used in NF and BF studies (Gruzelier 2014a; Rokicki et al. 1997) and should therefore be measured in further evaluations of this protocol. According to the proposed guidelines on reporting NF studies, future studies should register, analyze, and report regulation success, based on the feedback signal itself, as well as the detailed courses of changes in the trained as well as associated EEG parameters within and between sessions (Ros et al. 2020).

Further, we focused on the analysis of EEG changes on the position of the training electrode Cz. However, neurofeedback might also lead to topographic changes in brain activity and can result in significant alterations of EEG activity on positions not involved in the training (Gruzelier 2014a). Therefore, future studies with increased statistical power should analyze multiple EEG positions to assess possible topographic EEG changes.

Apart from limitations, our study has the strengths of analyzing objective physiological regulatory abilities with sophisticated EEG equipment and proper artifact corrections (Muthukumaraswamy 2013; Wallstrom, et al. 2004), in a standardized experimental design using reliable methods.

The experimental setup of the study implies another strength: We measured the EEG in the absence of feedback. Through this setup, we found that the down-regulation of cortical arousal was no longer dependent on provided feedback. These findings indicate that NF participants should be able to control their cortical arousal in everyday situations that include tempting confrontations with food cues (e.g., at dinner parties or cafeteria buffets). Hence, our results show a transfer process that accounts for external validity and effectiveness of the NF (Sherlin et al. 2011).

Evidence on physiological learning was mixed in prior research on BF and NF protocols (Imperatori et al. 2018). To our best knowledge, our study provides the first available insights into mechanisms in a high beta NF protocol with food cue exposure to reduce binge eating. Overall, the present results contribute to the body of evidence for the role of physiological changes associated with NF treatments that is heavily demanded by NF researchers (Bagdasaryan and Le Van Quyen, 2013; Gruzelier 2014a; Niv 2013; Ros et al. 2020; Sitaram et al. 2017; Strehl 2014).

Altogether, these results contribute to the notion that self-control abilities regarding the ‘flesh’ (i.e., physiological changes) can help increase the ‘spirit’s’ ability to resist temptation, showing that NF indeed can provide specific physiological contributions to change dysfunctional eating.

## References

[CR1] Adam TC, Epel ES (2007). Stress, eating and the reward system. Physiology & Behavior.

[CR2] Ali SS, Lifshitz M, Raz A (2014). Empirical neuroenchantment: From reading minds to thinking critically. Frontiers in Human Neuroscience.

[CR3] Bagdasaryan J, Le Van Quyen M (2013). Experiencing your brain: Neurofeedback as a new bridge between neuroscience and phenomenology. Frontiers in Human Neuroscience.

[CR4] Bartholdy S, Musiat P, Campbell IC, Schmidt U (2013). The potential of neurofeedback in the treatment of eating disorders: A review of the literature. European Eating Disorders Review.

[CR5] Blanchard EB, Schwarz SP (1988). Clinically significant changes in behavioral medicine. Behavioral Assessment.

[CR6] Blume M, Schmidt R, Hilbert A (2019). Abnormalities in the EEG power spectrum in bulimia nervosa, binge-eating disorder, and obesity: A systematic review. European Eating Disorders Review.

[CR7] Boswell RG, Kober H (2016). Food cue reactivity and craving predict eating and weight gain: A meta-analytic review. Obesity Reviews.

[CR8] Craven MP, Fekete EM (2019). Weight-related shame and guilt, intuitive eating, and binge eating in female college students. Eating behaviors.

[CR9] Colloca L, Miller FG (2011). The nocebo effect and its relevance for clinical practice. Psychosomatic Medicine.

[CR10] Dalton B, Campbell IC, Schmidt U (2017). Neuromodulation and neurofeedback treatments in eating disorders and obesity. Current Opinion in Psychiatry.

[CR11] Dinkel A, Berth H, Exner C, Rief W, Balck F (2005). Deutsche Adaptation der Restraint Scale zur Erfassung gezügelten Essverhaltens. Diagnostica.

[CR12] Dulloo AG, Montani JP (2015). Pathways from dieting to weight regain, to obesity and to the metabolic syndrome: An overview. Obesity Reviews.

[CR13] Egner T, Gruzelier JH (2001). Learned self-regulation of EEG frequency components affects attention and event-related brain potentials in humans. NeuroReport.

[CR14] Epstein LH, Blanchard EB (1977). Biofeedback, self-control, and self-management. Biofeedback and Self-regulation.

[CR15] Frank S, Lee S, Preissl H, Schultes B, Birbaumer N, Veit R (2012). The obese brain athlete: Self-regulation of the anterior insula in adiposity. PLoS ONE.

[CR16] Fritz CO, Morris PE, Richler JJ (2012). Effect size estimates: Current use, calculations, and interpretation. Journal of Experimental Psychology: General.

[CR17] Gruzelier JH (2014). EEG-neurofeedback for optimising performance. III: A review of methodological and theoretical considerations. Neuroscience & Biobehavioral Reviews.

[CR18] Gruzelier JH (2014). EEG-neurofeedback for optimising performance. I: A review of cognitive and affective outcome in healthy participants. Neuroscience & Biobehavioral Reviews.

[CR19] Haedt-Matt AA, Keel PK (2011). Revisiting the affect regulation model of binge eating: A meta-analysis of studies using ecological momentary assessment. Psychological Bulletin.

[CR20] Hilbert, A., & Tuschen-Caffier, B. (2006). *Eating Disorder Examination. Deutschsprachige Übersetzung*. Münster: Verlag für Psychotherapie, PAG Institut für Psychologie AG.

[CR21] Holroyd KA, Penzien DB, Hursey KG, Tobin DL, Rogers L, Holm JE, Marcille PJ, Hall JR, Chila AG (1984). Change mechanisms in EMG biofeedback training: Cognitive changes underlying improvements in tension headache. Journal of Consulting and Clinical Psychology..

[CR22] Ihssen N, Sokunbi MO, Lawrence AD, Lawrence NS, Linden DE (2017). Neurofeedback of visual food cue reactivity: A potential avenue to alter incentive sensitization and craving. Brain Imaging and Behavior.

[CR23] Imperatori C, Mancini M, Della Marca G, Valenti EM, Farina B (2018). Feedback-based treatments for eating disorders and related symptoms: A systematic review of the literature. Nutrients.

[CR24] Imperatori C, Valenti EM, Della Marca G, Amoroso N, Massullo C, Carbone GA (2017). Coping food craving with neurofeedback. Evaluation of the usefulness of alpha/theta training in a non-clinical sample. International Journal of Psychophysiology.

[CR25] Jasper HH (1958). The ten twenty electrode system of the international federation. Electroencephalography and Clinical Neurophysiology.

[CR26] Keith JR, Rapgay L, Theodore D, Schwartz JM, Ross JL (2015). An assessment of an automated EEG biofeedback system for attention deficits in a substance use disorders residential treatment setting. Psychology of Addictive Behaviors.

[CR27] Kemps E, Tiggemann M (2007). Modality-specific imagery reduces cravings for food: An application of the elaborated intrusion theory of desire to food craving. Journal of Experimental Psychology: Applied.

[CR28] Kim DK, Rhee JH, Kang SW (2014). Reorganization of the brain and heart rhythm during autogenic meditation. Frontiers in Integrative Neuroscience.

[CR29] Knäuper B, Pillay R, Lacaille J, McCollam A, Kelso E (2011). Replacing craving imagery with alternative pleasant imagery reduces craving intensity. Appetite.

[CR30] Lackner N, Unterrainer HF, Skliris D, Shaheen S, Dunitz-Scheer M, Wood G (2016). EEG neurofeedback effects in the treatment of adolescent anorexia nervosa. Eating disorders.

[CR31] La Vaque TJ, Hammond DC, Trudeau D, Monastra V, Perry J, Lehrer P (2002). Template for developing guidelines for the evaluation of the clinical efficacy of psychophysiological interventions. Applied psychophysiology and biofeedback.

[CR32] Leong SL, Vanneste S, Lim J, Smith M, Manning P, De Ridder D (2018). A randomised, double-blind, placebo-controlled parallel trial of closed-loop infraslow brain training in food addiction. Scientific Reports.

[CR33] Meule A, Freund R, Skirde AK, Vögele C, Kübler A (2012). Heart rate variability biofeedback reduces food cravings in high food cravers. Applied Psychophysiology and Biofeedback.

[CR34] Mond JM, Hay PJ, Rodgers B, Owen C (2006). Eating Disorder Examination Questionnaire: Norms for young adult women. Behavior Research and Therapy.

[CR35] Muthukumaraswamy SD (2013). High-frequency brain activity and muscle artifacts in MEG/EEG: A review and recommendations. Frontiers in Human Neuroscience.

[CR36] Niv S (2013). Clinical efficacy and potential mechanisms of neurofeedback. Personality and Individual Differences.

[CR37] Ozier AD, Kendrick OW, Leeper JD, Knol LL, Perko M, Burnham J (2008). Overweight and obesity are associated with emotion-and stress-related eating as measured by the eating and appraisal due to emotions and stress questionnaire. Journal of the American Dietetic Association.

[CR38] Paquette V, Beauregard M, Beaulieu-Prévost D (2009). Effect of a psychoneurotherapy on brain electromagnetic tomography in individuals with major depressive disorder. Psychiatry Research: Neuroimaging.

[CR39] Parvaz MA, Alia-Klein N, Woicik PA, Volkow ND, Goldstein RZ (2011). Neuroimaging for drug addiction and related behaviors. Reviews in the Neurosciences.

[CR40] Presnell K, Bearman SK, Stice E (2004). Risk factors for body dissatisfaction in adolescent boys and girls: A prospective study. International Journal of Eating Disorders.

[CR41] Rokicki LA, Holroyd KA, France CR, Lipchik GL, France JL, Kvaal SA (1997). Change mechanisms associated with combined relaxation/EMG biofeedback training for chronic tension headache. Applied Psychophysiology and Biofeedback.

[CR42] Ros T, Enriquez-Geppert S, Zotev V, Young KD, Wood G, Whitfield-Gabrieli S (2020). Consensus on the reporting and experimental design of clinical and cognitive-behavioural neurofeedback studies (CRED-nf checklist). Brain.

[CR43] Rostami R, Sadeghi H, Karami KA, Abadi MN, Salamati P (2012). The effects of neurofeedback on the improvement of rifle shooters' performance. Journal of Neurotherapy.

[CR44] Schmidt J, Martin A (2015). Neurofeedback reduces overeating episodes in female restrained eaters—A randomized controlled pilot-study. Applied Psychophysiology and Biofeedback.

[CR45] Schmidt J, Martin A (2016). Neurofeedback against binge eating: A randomized controlled trial in a female subclinical threshold sample. European Eating Disorders Review.

[CR46] Schwartz GE (1976). Self-regulation of response patterning. Biofeedback and Self-Regulation.

[CR47] Schwartz MS, Andrasik F, Schwartz MS, Andrasik F (2003). Evaluation research in clinical biofeedback. Biofeedback—A Practitioner’s Guide.

[CR48] Shapiro D, Crider AB, Tursky B (1964). Differentiation of an autonomic response through operant reinforcement. Psychonomic Science.

[CR49] Sherlin LH, Arns M, Lubar J, Heinrich H, Kerson C, Strehl U, Sterman MB (2011). Neurofeedback and basic learning theory: Implications for research and practice. Journal of Neurotherapy.

[CR50] Siniatchkin M, Kropp P, Gerber WD (2000). Neurofeedback−the significance of reinforcement and the search for an appropriate strategy for the success of self-regulation. Applied Psychophysiology and Biofeedback.

[CR51] Sitaram R, Ros T, Stoeckel L, Haller S, Scharnowski F, Lewis-Peacock J (2017). Closed-loop brain training: the science of neurofeedback. Nature Reviews Neuroscience.

[CR52] Skinner HH, Haines J, Austin SB, Field AE (2012). A prospective study of overeating, binge eating, and depressive symptoms among adolescent and young adult women. Journal of Adolescent Health.

[CR53] Sobik L, Hutchison K, Craighead L (2005). Cue-elicited craving for food: A fresh approach to the study of binge eating. Appetite.

[CR54] Strehl U (2014). What learning theories can teach us in designing neurofeedback treatments. Frontiers in Human Neuroscience.

[CR55] Stroebe W, Mensink W, Aarts H, Schut H, Kruglanski AW (2008). Why dieters fail: Testing the goal conflict model of eating. Journal of Experimental Social Psychology.

[CR56] Swinburn BA, Sacks G, Hall KD, McPherson K, Finegood DT, Moodie ML, Gortmaker SL (2011). The global obesity pandemic: Shaped by global drivers and local environments. The Lancet.

[CR57] Teufel M, Stephan K, Kowalski A, Käsberger S, Enck P, Zipfel S, Giel KE (2013). Impact of biofeedback on self-efficacy and stress reduction in obesity: A randomized controlled pilot study. Applied Psychophysiology and Biofeedback.

[CR58] Thibault RT, Lifshitz M, Raz A (2016). The self-regulating brain and neurofeedback: Experimental science and clinical promise. Cortex.

[CR59] Thibault RT, Lifshitz M, Raz A (2017). Neurofeedback or neuroplacebo?. Brain.

[CR60] Thibault RT, Lifshitz M, Raz A (2018). The climate of neurofeedback: scientific rigour and the perils of ideology. Brain.

[CR61] Wallstrom GL, Kass RE, Miller A, Cohn JF, Fox NA (2004). Automatic correction of ocular artifacts in the EEG: A comparison of regression-based and component-based methods. International Journal of Psychophysiology.

[CR62] Welch PD (1967). The use of fast Fourier transform for the estimation of power spectra: A method based on time averaging over short, modified periodograms. IEEE Transactions on Audio and Electroacoustics.

[CR63] Wickramasekera I (1999). How does biofeedback reduce clinical symptoms and do memories and beliefs have biological consequences? Toward a model of mind-body healing. Applied Psychophysiology and Biofeedback.

[CR64] Zotev V, Phillips R, Yuan H, Misaki M, Bodurka J (2014). Self-regulation of human brain activity using simultaneous real-time fMRI and EEG neurofeedback. NeuroImage.

